# Unscrambling Cyanobacteria Community Dynamics Related to Environmental Factors

**DOI:** 10.3389/fmicb.2016.00625

**Published:** 2016-05-09

**Authors:** Mireia Bertos-Fortis, Hanna M. Farnelid, Markus V. Lindh, Michele Casini, Agneta Andersson, Jarone Pinhassi, Catherine Legrand

**Affiliations:** ^1^Department of Biology and Environmental Science, Centre for Ecology and Evolution in Microbial Model Systems, Linnaeus UniversityKalmar, Sweden; ^2^Swedish University of Agricultural Sciences, Department of Aquatic Resources, Institute of Marine ResearchLysekil, Sweden; ^3^Department of Ecology and Environmental Sciences, Umeå UniversityUmeå, Sweden

**Keywords:** cyanobacteria, community, environmental factors, climate change, temperature, salinity

## Abstract

Future climate scenarios in the Baltic Sea project an increase of cyanobacterial bloom frequency and duration, attributed to eutrophication and climate change. Some cyanobacteria can be toxic and their impact on ecosystem services is relevant for a sustainable sea. Yet, there is limited understanding of the mechanisms regulating cyanobacterial diversity and biogeography. Here we unravel successional patterns and changes in cyanobacterial community structure using a 2-year monthly time- series during the productive season in a 100 km coastal-offshore transect using microscopy and high-throughput sequencing of 16S rRNA gene fragments. A total of 565 cyanobacterial OTUs were found, of which 231 where filamentous/colonial and 334 picocyanobacterial. Spatial differences in community structure between coastal and offshore waters were minor. An “epidemic population structure” (dominance of asingle cluster) was found for Aphanizomenon/Dolichospermum within the filamentous/colonial cyanobacterial community. In summer, this clusters imultaneously occurred with opportunistic clusters/OTUs, e.g., Nodularia spumigena and Pseudanabaena. Picocyanobacteria, Synechococcus/Cyanobium, formeda consistent but highly diverse group. Overall, the potential drivers structuring summer cyanobacterial communities were temperature and salinity. However, the different responses to environmental factors among and within genera suggest high niche specificity for individual OTUs. The recruitment and occurrence of potentially toxic filamentous/colonial clusters was likely related to disturbance such as mixing events and short-term shifts in salinity, and not solely dependent on increasing temperature and nitrogen-limiting conditions. Nutrients did not explain further the changes in cyanobacterial community composition. Novel occurrence patterns were identified as a strong seasonal succession revealing a tight coupling between the emergence of opportunistic picocynobacteria and the bloom offilamentous/colonialclusters. These findings highlight that if environmental conditions can partially explain the presence of opportunistic picocyanobacteria, microbial and trophic interactions with filamentous/colonial cyanobacteria should also be considered as potential shaping factors for single-celled communities. Regional climate change scenarios in the Baltic Sea predict environmental shifts leading to higher temperature and lower salinity; conditions identified here as favorable for opportunistic filamentous/colonial cyanobacteria. Altogether, the diversity and complexity of cyanobacterial communities reported here is far greater than previously known, emphasizing the importance of microbial interactions between filamentous and picocyanobacteria in the context of environmental disturbances.

## Introduction

One of the major challenges for the scientific community and environmental managers is to understand and project the effects of both climate change and human activities on biogeochemical cycles in aquatic systems. Phytoplankton are key organisms in marine ecosystems, converting inorganic carbon into organic matter through photosynthesis, and thereby providing the reduced organic carbon that fuels the entire food web, from zooplankton to top predators. Filamentous cyanobacteria are major components of the phytoplankton community in the eutrophic waters of the Baltic Sea, contributing more than 50% of the total primary production of the cyanobacterial summer bloom (Stal et al., 2003). This functional group of organisms shows extreme plasticity toward changes in environmental conditions and has been recorded yearly in summer months for ca. 7000 years (estimated from sediment cores; Bianchi et al., 2000). In addition, the potential toxicity of specific cyanobacteria can compromise other trophic levels, as cyanobacterial hepatotoxins are specific inhibitors of serine/threonine protein phosphatases and tumor promoters (Eriksson et al., 1990; Ohta et al., 1994). These toxins, nodularins and microcystins, produced by *Nodularia spumigena*, *Dolichospermum* and *Microcystis* spp. have negative effects on ecosystem services like fish production, hence affecting sustainability of water bodies (Karjalainen et al., 2007). During the last decades, there has been an increase in the magnitude and duration of cyanobacterial blooms (Kahru and Elmgren, 2014), which can be attributed to increasing anthropogenic eutrophication (Larsson et al., 1985; Zillén and Conley, 2010) and climate change (Paerl and Huisman, 2009).

Climate change scenarios are uncertain in terms of particular effects in space and time at local and regional scales. Nevertheless, there are clear indications for effects altering global marine ecosystems (Hoegh-Guldberg and Bruno, 2010). Predicted shifts in environmental conditions due to climate change in the Baltic Sea include higher temperature, increased precipitation and consequently higher river run-off and lower salinities (Meier et al., 2014). Recent climate change models have introduced these environmental projections on the dynamics of Baltic Sea cyanobacteria (Hense et al., 2013). Results show an increase in biomass in 30 years with an earlier onset of the summer bloom. Still, it is currently not possible to explain conclusively why surface accumulations of cyanobacteria occur 3 weeks earlier today than four decades ago (Kahru and Elmgren, 2014). Calmer weather, higher temperature, distance to the shore, and changes in the dominant species within cyanobacterial community are potential factors to explain that cyanobacteria float to the surface earlier or more often. At the moment, there is little understanding of the mechanisms regulating changes in cyanobacterial community composition, which will progressively gain importance given the shifts in environmental conditions due to climate change.

Cyanobacteria are mainly studied during summer in the Baltic Sea, the season in which filamentous and colonial cyanobacteria dominate the phytoplankton community due to their ability to fix atmospheric nitrogen at low nitrogen (N) to phosphorus (P) ratios (Niemi, 1979). The main species forming the summer cyanobacterial blooms are *Aphanizomenon* sp., *N. spumigena* and the revised genus *Dolichospermum* sp. – formerly *Anabaena* sp. (Wacklin et al., 2009). Lower temperature, reduced salinity and irradiance favor *Aphanizomenon* sp., while *N. spumigena* prefers higher temperature and irradiance (Stal et al., 2003). *Aphanizomenon* can be found in the water column throughout the year, while *N. spumigena* and *Dolichospermum* are mainly found in summer (Suikkanen et al., 2010). Unicellular cyanobacteria (picocyanobacteria) are present in Baltic waters all year round and their seasonal dynamics are often analyzed in conjunction with heterotrophic bacterioplankton assemblages (Andersson et al., 2010; Herlemann et al., 2011; Dupont et al., 2014; Lindh et al., 2015).

Conventional taxonomic classification of filamentous and colonial cyanobacteria has been based on morphology, but this classification is often revised through phylogenetic analyses based on molecular sequence data (Komárek et al., 2014). Molecular analyses have addressed phylogeny focusing on specific species/genera at a time, e.g., *N. spumigena* or *Aphanizomenon/Dolichospermum* (Barker et al., 1999; Lyra et al., 2001; Gugger et al., 2002). However, such molecular analyses are not directly informative about the morphological diversity of filamentous and colonial cyanobacteria due to their high sequence identity in 16S rRNA (e.g., Gugger et al., 2002). Picocyanobacteria, on the other hand, are small cells and their taxonomic affiliation is hardly distinguishable under microscopy (Waterbury et al., 1986), which makes molecular approaches crucial to distinguish among species. Picocyanobacteria are a very diverse phylogenetic group with multiple genetic lineages, for which community dynamics have been extensively studied in marine ecosystems (Urbach et al., 1998; Haverkamp et al., 2008; Ahlgren and Rocap, 2012; Larsson et al., 2014). Overall, these studies rarely report community composition data for all types of cyanobacteria. Therefore, combined studies (genetic and morphological diversity) including both filamentous/colonial cyanobacteria and picocyanobacteria are necessary to resolve the diversity and biogeography of cyanobacteria.

Recent advances in high-throughput sequencing now allows for the study of both filamentous/colonial and picocyanobacteria with concurrent morphological approaches. In this study we aimed to investigate the spatial and temporal dynamics of cyanobacterial communities in the upper mixed layer (10 m) of the Baltic Sea by applying pyrosequencing V3–V4 of the 16S rRNA gene coupled with microscopy analysis. Additionally, we addressed the potential role of principal environmental variables triggering seasonal changes in cyanobacterial communities, specifically for potential toxic genera such as *Nodularia* and *Dolichospermum*.

## Materials and Methods

### Sampling Location and Sample Collection

Water samples were collected along a coastal-offshore transect located in the northern Kalmar strait and the southern Western Gotland Sea, in the Central Baltic Sea. During the 2-year survey (2010–2011), 16 stations were sampled monthly covering the productive period (April–October, stations PF1-16, **Supplementary Figure S1** modified from Legrand et al., 2015). Temperature and salinity data were collected using a CTD probe (AAQ1186-H, Alec Electronics, Japan) and averaged for the first 10 m. For all samples, water from 2, 4, 6, 8, and 10 m depth was pooled into acid-washed and Milli-Q-rinsed polycarbonate bottles. Chlorophyll *a* (Chl *a*), used as proxy for phytoplankton biomass, was measured fluorometrically after ethanol extraction (Jespersen and Christoffersen, 1987). Samples for heterotrophic bacterial abundance were preserved in 2% formaldehyde, kept at -80°C and analyzed using flow cytometry (BD FACs Calibur) using SYTO13 (Gasol and del Giorgio, 2000). Samples for nutrients were taken in 2011, GF/C filtered and analyzed using colorimetric methods according to Valderrama (1995).

The sampling stations were classified as coastal, intermediate or offshore based on bathymetry and distance to the coastline (for details see Legrand et al., 2015). The study was part of a large-scale field experiment, within the PLAN FISH project, investigating ecosystem responses and dynamics to reducing planktivores over 2010–2012.

### Filamentous/Colonial Cyanobacteria and Other Phytoplankton Enumeration

A total of 240 samples were screened under microscopy, for filamentous/colonial cyanobacteria and other phytoplankton. Water samples were preserved in 2% Lugol solution and stored in the dark at room temperature until further examination. Subsamples were transferred into sedimentation chambers (10 ml) for approximately 24 h before counting with an Olympus CKX 41 inverted light microscope. In each sample a minimum of 300 cells were counted (*SD* ≤ 11%). Phytoplankton cells including filamentous and colonial cyanobacteria were identified to genus and species level whenever possible. Morphological criteria for cyanobacteria were filament (trichome) length and shape, width and length of cells, presence of heterocysts, and presence and shape of akinetes. Taxonomical confirmation was achieved by consulting the database Nordic Microalgae^1^ validated by the HELCOM Phytoplankton Expert Group. Cell biomass was calculated from biovolume (Olenina et al., 2006) and carbon content (Edler, 1979).

### Community DNA Extraction, PCR Amplification and 454-Pyrosequencing

The sample collection from 2010 included coastal, intermediate and offshore stations while samples from 2011 included only coastal and offshore stations. Seawater (1 L) was filtered onto a 0.2 μm Supor Filter (47 mm, PALL corporation). The filters were placed in individual cryovials, supplemented with 1 mL TE buffer (10 mM Tris, 1 mM EDTA, pH 8.0) and stored at -80°C until extraction. Community DNA was extracted using an enzyme/phenol-chloroform protocol (Riemann et al., 2000). In total, 118 samples were selected for 454-pyrosequencing. The V3–V4 hypervariable region of the bacterial 16S rRNA gene was amplified by using primers 341F and 805R as described in Herlemann et al. (2011). The 16S rRNA gene amplicons were quantified with nanodrop, pooled at equimolar amounts and sequenced using the Roche GS-FLX 454 automated pyrosequencer (Roche Applied Science, Branford, CT, USA) at SciLifeLab, Stockholm (Sweden). Samples from each year were sequenced on separate 454 plates resulting in 300000 reads from 2010 and 396000 reads from 2011 with an average read length of 350 bp. Denoising and screening for chimera removal was performed following Quince et al. (2011). The reads were clustered at 98% identity by applying UCLUST (Edgar, 2010). A total of 12,636 Archaea, cyanobacteria, and other bacteria OTUs were identified (excluding singletons). The level of clustering (98%) was selected to reduce 454 data noise levels. Likely at this cut-off, different species with high 16S rRNA gene identity could fall into the same cluster and microdiversity might be underestimated. In cyanobacteria, high 16S rRNA gene identity has been described for *Aphanizomenon* and *Dolichospermum* and these species are therefore not represented by individual OTUs. Rarefaction curves at 98% clustering showed saturation in all the samples indicating sufficient sampling effort covering cyanobacterial diversity (**Supplementary Figure S2**). The 98% rarefaction curves show an estimated maximum richness of 5–30 OTU in samples collected during the survey. Normalization of sequence reads was performed by dividing the number of reads of each OTU within a sample by the total reads from that specific sample. Cyanobacterial OTUs or clusters were characterized as generalists if they were detected in all samples and at all stations; and as opportunists when OTUs/clusters occurred only occasionally, sporadically or seasonally. DNA sequences can be found in the National Center for Biotechnology Information (NCBI) Sequence Read Archive under accession number SRP023607.

### Phylogenetic Analyses and Correlations to Environmental Factors

Taxonomic identification was done using the SINA/SILVA database and unclassified OTUs were resolved using NCBI blastn. The cyanobacterial OTUs (565 in total) were classified phylogenetically as filamentous/colonial cyanobacteria (231 OTUs) or picocyanobacteria (334 OTUs). Partial least squares (PLS) regression was used to describe the relationships between community composition (Y) and environmental variables X (e.g., temperature, salinity). The ability of the PLSr to find reliable latent variables (PLSr components) is affected by the n^∗^p and m^∗^p dimensions of X and Y matrices, respectively. In our situation the high number of OTUs relative to the small number of sites (m >> p) did not allow us to find reliable latent variables for Y. To remedy this, we considered only the most abundant OTUs. The *R*^2^s (*R*^2^*Y* and *R*^2^*X*) were used to evaluate explanatory power and fit of the model. However, since we used PLSr mainly for describing the relationships between cyanobacterial community composition and environmental variables, we were less interested in optimizing the predictive power of the model assessed by the *Q*^2^. PLS analyses were run with package plsdepot (Sanchez, 2015).

We also run PERMANOVA, to confirm patterns detected with the PLS. We assessed betadispersion, which is the underlying assumption to perform PERMANOVA test. PERMANOVA analyses (999 permutations) were run to relate differences in Bray Curtis dissimilarity matrix of the cyanobacterial community composition to temperature and salinity. Vegan package was used to perform PERMANOVA (Oksanen et al., 2016).

A maximum likelihood phylogenetic tree of the 50 OTUs with the highest relative abundance in 2010 and 2011, respectively, corresponding to 72 OTUs in total, was created in MEGA 6 (Tamura et al., 2013). Relative abudances of these 72 most abundant OTUs were visualized using a heatmap. As the assumption of normality was not met in our dataset after transformation, Spearman correlations (ρ) were used to find relationships between cyanobacterial OTU occurrence and abiotic/abiotic factors. Resulting Spearman coefficients were plotted in a heatmap. All statistical analyses were performed in R studio 0.98.945.

## Results

### Environmental Dynamics

Water temperature was 1–2.5°C in early spring and increased to 17–20°C in summer and fall (**Supplementary Figure S3**). Salinity was slightly lower in 2010 (6.00–6.82) compared to 2011 (6.15–7.20, **Supplementary Figure S3**). In July–August of both years, a decrease in salinity (up to -0.60 units) was observed, with lowest values recorded at the offshore stations. Nutrient dynamics displayed substantial seasonal variation with maximum TN (20–30 μM) in summer and maximum TP (1.0–1.2 μM) in late fall (**Supplementary Figure S3**). Consequently, the TN:TP ratio = 40 (>Redfield ratio N:P = 16) showed that P was the limiting element rather than N for most of the year, except during fall (**Supplementary Figure S3**). Dissolved inorganic nitrogen (DIN: 0.2–4.0 μM) and phosphate (0.07–0.60 μM) showed lowest values during summer (**Supplementary Figure S3**). In both years, there was a distinct spring bloom at low temperature composed of diatoms and dinoflagellates (Legrand et al., 2015). Chlorophyll *a* levels were twice as high in spring (7–8 μg Chl *a* L^-1^) compared to summer (2–3 μg Chl *a* L^-1^) when cyanobacteria bloomed (**Supplementary Figure S3**).

### Cyanobacteria Seasonal Dynamics

#### Richness and Biomass Determination from Microscopy Observations

**FIGURE 1:**
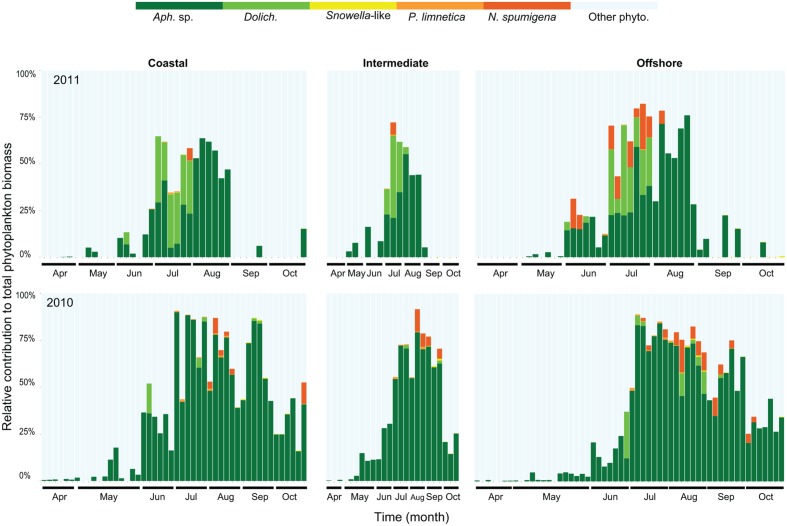
**Relative contribution (carbon content) of filamentous and colonial cyanobacterial species to the total phytoplankton biomass in coastal, intermediate and offshore stations from 2010 to 2011.** Abbreviations correspond to *Aphanizomenon* sp. (*Aph.* sp.), *Dolichospermum* (*Dolich.*), *Pseudanabaena limnetica* (*P. limnetica*), *Nodularia spumigena* (*N. spumigena*), and other phytoplankton (other phyto.). Each bar represents one station and sampling occasion, 128 samples in 2010 and 112 samples in 2011.

The seasonal changes in cyanobacterial community composition in the upper mixed layer were similar at all sampled stations (**Figure 1** modified from Legrand et al., 2015). The contribution of filamentous and colonial cyanobacteria to the annual phytoplankton biomass was higher in 2010 (34%) compared to 2011 (12%; Legrand et al., 2015). The filamentous *Aphanizomenon* was the most common genus reaching maximum biomass (75–90% of the total phytoplankton biomass) in July-August (**Figure 1**). *Dolichospermum*, *Pseudanabaena*, and *Nodularia* were also found in summer months with a larger combined contribution of the total phytoplankton biomass in July 2011 (up to 50%) compared to July 2010 (15% of the total phytoplankton biomass). Lower abundances of *Pseudanabaena* and *Snowella-*like species (colonial) were occasionally observed (7.79 mm mL^-1^ and 2600 cells mL^-1^, respectively) mostly during summer months. The duration of the cyanobacterial bloom was shorter in 2011 (3 months) compared to 2010 (5 months), in which *Aphanizomenon* sp. and *N. spumigena* showed an extended bloom season from early summer to late fall.

#### 16S rRNA Gene Phylogenetic Analysis: Richness and Occurrence

**FIGURE 2:**
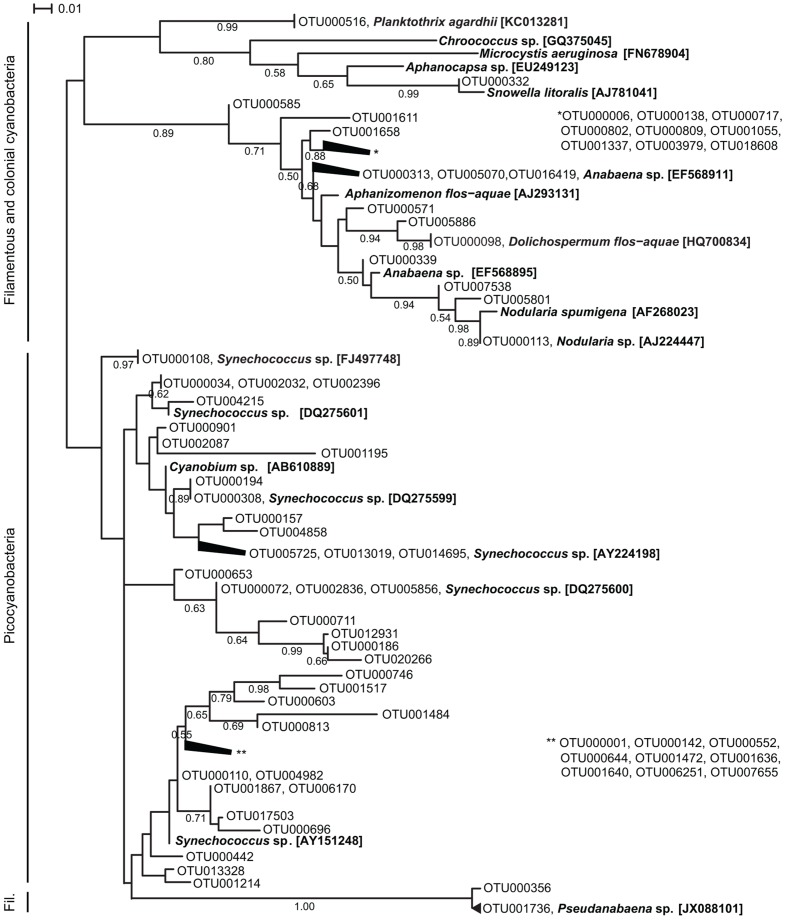
**Maximum likelihood tree based on representative sequences of OTUs (98% identity) of partial 16S rRNA gene sequences (cut to 350 bp long).** For clarity, the 50 most abundant OTUs for 2010 and 2011, respectively (72 OTUs in total) are shown in the tree and branches of <0.01 distance have been collapsed. Reference sequences were retrieved from NCBI and their accession numbers are shown in brackets. Branch lengths were determined using the Tamura-Nei model, bootstrap values were calculated (1000 replicate trees) and are displayed when greater than 0.50.

**FIGURE 3:**
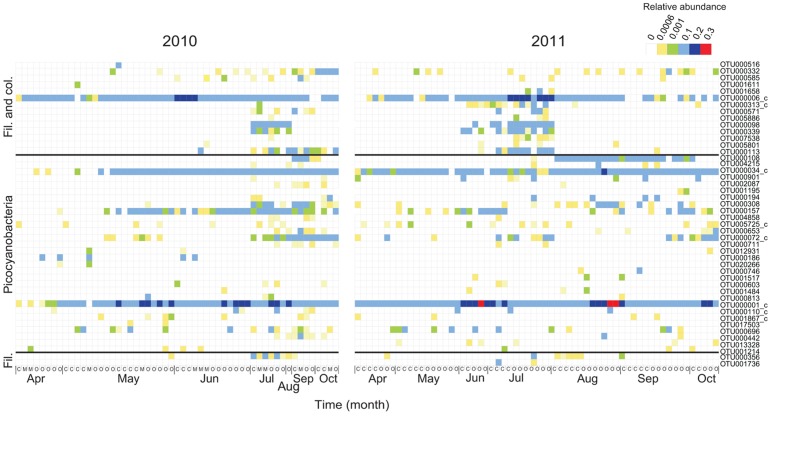
**Temporal and spatial dynamics of the 50 most abundant cyanobacterial OTUs (98% identity) for 2010 and 2011, respectively (72 OTUs in total).** The order of the OTUs corresponds to **Figure 2** and OTUs with <0.01 phylogenetic distance (350 bp long sequences) have been clustered (denoted with c). The colors in the heatmap represent the relative abundance of each OTU in a specific sample. The month of sampling and the classification of each station, coastal (C), intermediate (M), and open (O) are indicated on the *X*-axis.

The OTU richness of filamentous and colonial cyanobacteria, defined by the number of OTUs, was low during both spring and fall (<10 OTUs) and highest in July for both years (15–37 OTUs; **Supplementary Figure S4**). Phylogenetically, the cyanobacterial OTUs formed distinct clusters corresponding to morphology (filamentous, colonial, single cell) and function (heterocystous, non-heterocystous; **Figure 2**). The dominating *Aphanizomenon/Dolichospermum* phylotype (OTU000006, **Supplementary Figure S5A**) was 100% similar to a freshwater *Aphanizomenon* strain (HG917867; Casero et al., 2014). In summer months, there was an increase of opportunistic OTUs where *Aphanizomenon/Dolichospermum*, *Nodularia*, and *Pseudanabaena* clusters were frequently detected (**Figure 3**). Several *Aphanizomenon/Dolichospermum* (e.g., OTU000098) phylotypes only occurred in summer, while a *Nodularia* phylotype sustained until October in 2010 (**Figure 3**). The most frequently detected *Nodularia* phylotype was closely related to an isolate from the Baltic Sea (100% identity; KF360086.1; Fewer et al., 2013). This *Nodularia* phylotype (OTU000113) showed high interannual variability with a higher relative abundance in 2011 compared to 2010 (**Figure 3** and **Supplementary Figure S5B**). Colonial cyanobacteria phylotype related to *Snowella* (99% identity) was present during both years and showed a patchy distribution.

Picocyanobacterial richness patterns were uniform over the sampling period for both years, with an increase toward summer when maximum richness was reached (>25 OTUs; **Supplementary Figure S4**). Picocyanobacterial OTUs were closely related to *Synechococcus* and *Cyanobium* (**Figure 2**) and were present at all times at all stations (**Figure 3**). The dominant phylotype (OTU000001) was 100% similar to a *Synechococcus* isolate from a subalpine lake (AY151250; Crosbie et al., 2003). This phylotype was present in all samples, reaching relative abundances up to 24% (**Supplementary Figure S5C**). Other *Synechococcus* OTUs (e.g., OTU00034 and OTU000072), showed more seasonal variability with higher relative abundances in late summer/fall than during spring (**Supplementary Figures S5D,E**). Their closest relatives were brackish and also saline water isolates (100% identity; DQ275607; and 100% identity; DQ275600; Sánchez-Baracaldo et al., 2008).

### Cyanobacterial Community Composition and Environmental Variables

**FIGURE 4:**
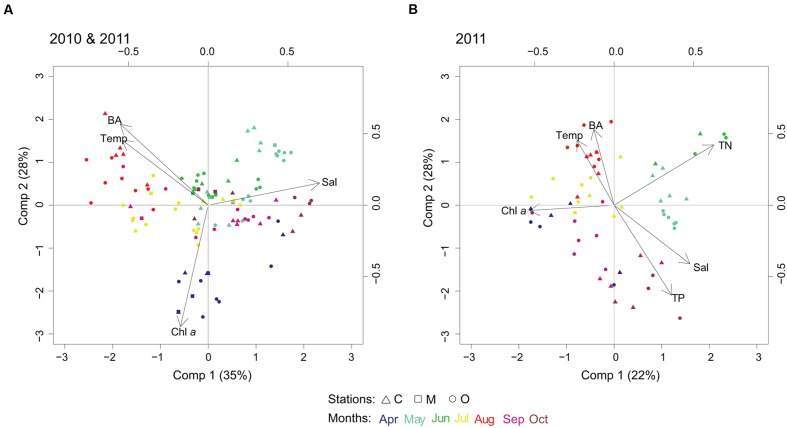
**Biplots resulting from partial least squares (PLS) regression model, linking cyanobacterial community composition with abiotic/biotic factors.** PLS biplots considering **(A)** temperature (Temp), salinity (Sal), chlorophyll *a* (Chl *a*), and heterotrophic bacterial abundance (BA), year 2010 and 2011, 100 most abundant cyanobacterial OTUs; and **(B)** temperature (Temp), salinity (Sal), chlorophyll *a* (Chl *a*), bacterial abundance (BA), total nitrogen (TN) and total phosphorus (TP), year 2011, 50 most abundant cyanobacterial OTUs.

The first PLS model was run with the 100 most abundant cyanobacterial OTUs for years 2010 and 2011, and temperature, salinity, Chl *a* and heterotrophic bacterial abundance as explanatory variables. Two components were selected by cross-validation for performing the PLS model (*R*^2^*Y* = 13%, *R*^2^*X* = 63%, see Supplementary Table S1A for *Q*^2^). Visual clustering of samples could be detected by month (**Figure 4**). Variations in spring cyanobacterial communities were linked to high Chl *a* and low temperature. In contrast, summer communities were related to high temperature and heterotrophic bacterial abundance together with low salinity (**Figure 4A**).

In the second PLS model, we added nutrients (TN and TP) to the previous set of explanatory variables (p = 6) for 2011 (50 most abundant OTUs, **Figure 4B**). Three components were selected by cross-validation for performing the PLS model (*R*^2^*Y* = 50%, *R*^2^*X* = 69%, see Supplementary Table S1B for *Q*^2^). Nutrients did not explain further the change in summer cyanobacterial communities that was dominated by filamentous and colonial genera, but high temperature and low salinity were potential factors describing community changes. The influence of geographic location in shaping the community composition (i.e., coastal, intermediate, offshore) showed unclear patterns compared to the impact of changes in environmental factors.

PERMANOVA tests results showed that cyanobacterial community composition was significantly different with temperature and salinity (see Supplementary Table S2).

**FIGURE 5:**
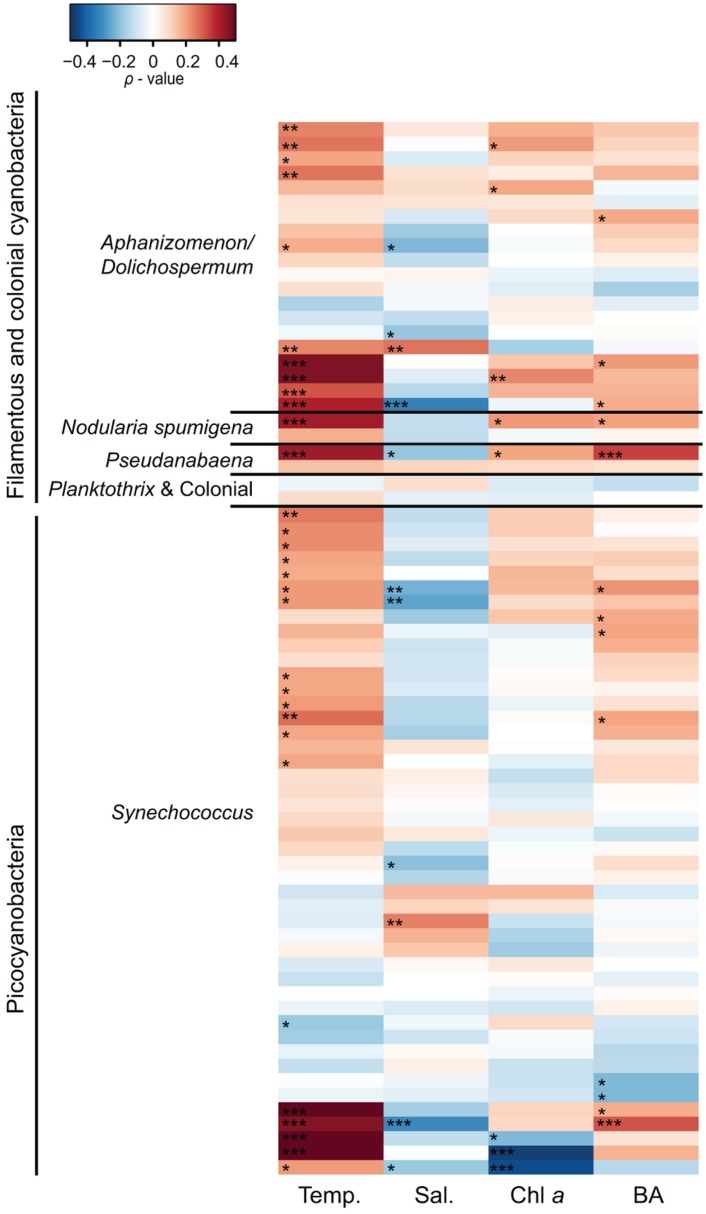
**Heatmap representing Spearman correlation coefficients between the 50 most abundant cyanobacterial OTUs in 2010 and 2011, respectively (n = 72) and environmental variables.** Temperature (Temp), salinity (Sal), chlorophyll *a* (Chl *a*), and bacterial abundance (BA) were used for the relationships. Phylotypes were classified according to their phylogenetic affiliations (**Figure 2**). Statistical significance is ^∗∗∗^*p* < 0.001, ^∗∗^*p* < 0.01, ^∗^*p* < 0.05.

**FIGURE 6:**
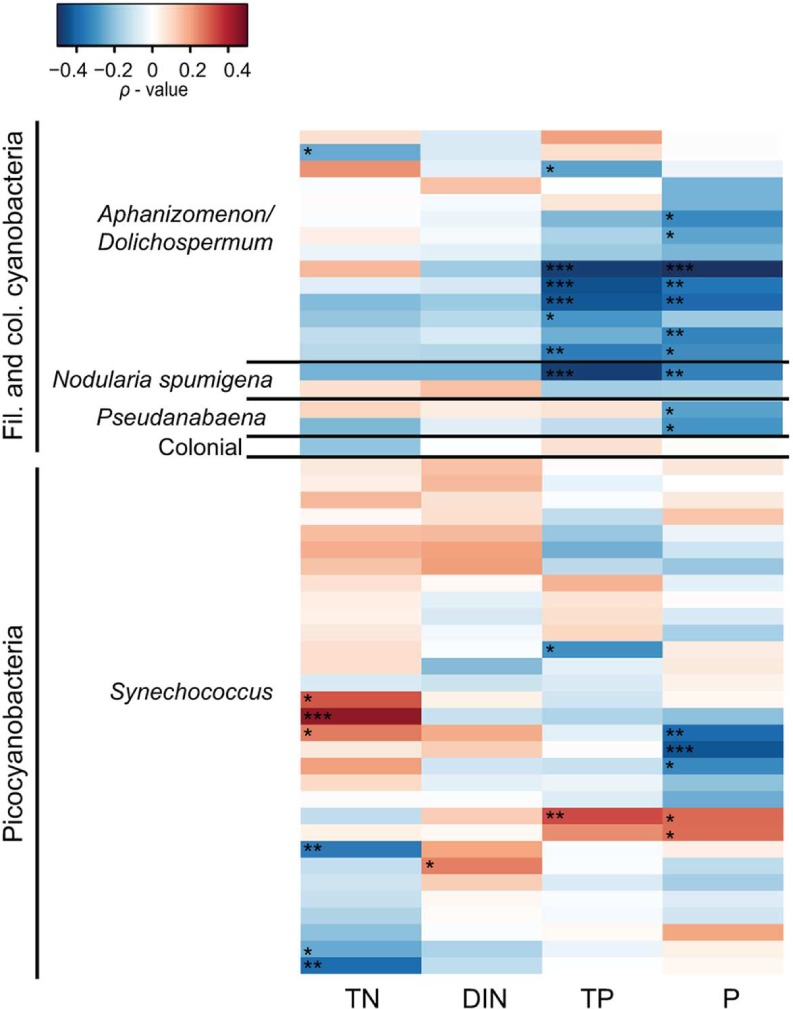
**Heatmap representing Spearman correlation coefficients between the 50 most abundant cyanobacterial OTUs in 2011 and nutrients.** Total nitrogen (TN), dissolved inorganic nitrogen (DIN; nitrogen and ammonium), total phosphorus (TP), phosphate (P) were used for the relationships. Only 2011 is shown because nutrient data was lacking for 2010. Statistical significance is ^∗∗∗^*p* < 0.001, ^∗∗^*p* < , ^∗^*p* < 0.05.

Spearman correlation analysis revealed high variability between and within cyanobacterial genera in response to different environmental parameters (**Figures 5** and **6**). In general, filamentous cyanobacteria showed a positive correlation with temperature (**Figure 5**). Many OTUs showed a significant positive correlation with increasing temperature (ρ_Temp_ > 0.3, *p* < 0.05). Moreover, some phylotypes affiliated with *Aphanizomenon/Dolichospermum*, *Nodularia*, and *Pseudanabaena* were negatively correlated to salinity (ρ_Sal_ < -0.3). Filamentous cyanobacteria showed positive correlation with Chl *a* and stronger positive relationships could be detected with heterotrophic bacterial abundance (ρ_BA_ > 0.2, *p* < 0.05). Overall, filamentous cyanobacteria were negatively correlated with TN, DIN, TP and P (**Figure 6**). For picocyanobacteria, many *Synechococcus* OTUs showed positive correlations with temperature and heterotrophic bacterial abundance (ρ_BA_ > 0.3, *p* < 0.05). However, salinity and Chl *a* had a negative impact on *Synechococcus* OTUs (ρ_Sal_ < -0.3 and ρ_Chl_ *_a_* < -0.4, *p* < 0.001). *Synechococcus* OTUs showed high variability in nutrient affinity, ranging from highly positive to highly negative correlations with TN, DIN, TP, and P. No significant relationship was found for other nutrients e.g., silica and ammonium and cyanobacterial OTUs (data not shown).

## Discussion

### Biomass and Distribution of Filamentous and Colonial Cyanobacteria

Massive blooms of filamentous/colonial cyanobacteria in the Baltic Sea Proper are a recurrent phenomenon in summer. The transport of P to surface layers, caused by oxygen depletion in bottom waters, is a vicious cycle promoting the occurrence of diazotrophic cyanobacteria (Vahtera et al., 2007). Whether they can thrive for longer periods in the pelagic zone is uncertain but their ability to fix N is of great advantage compared to picocyanobacteria in the planktonic habitat. Our data confirmed that cyanobacterial bloom intensity is highest during the summer months (**Figure 1**). However, we noted various cyanobacterial occurrence patterns in different genera/species and community dynamics. Interannual variation in magnitude and species composition of summer blooms can be considerable (Hajdu et al., 2007; Lips and Lips, 2008; Legrand et al., 2015). Blooms can persist up to one or 2 months in the Gulf of Finland and in the Southern Baltic Sea Proper (Lips and Lips, 2008; Suikkanen et al., 2010; Mazur-Marzec et al., 2013b). In the Baltic Sea Proper (this study), cyanobacterial blooms could exhibit their maximum during 3 to 5 months (June–August/October), supported by data of the annual phytoplankton biomass (Legrand et al., 2015). Different results in cyanobacterial bloom duration between studies can be explained by different sampling strategies i.e., integrated 0–10 m sample (this study) or discrete samples at 4–5 m (Gulf of Finland, South Baltic Proper).

In the upper mixed layer, *Aphanizomenon* colonies occurred at low abundance year-round and dominated the biomass during summer, while *Nodularia* and *Dolichospermum* filaments appeared in early summer (**Figure 1**). Such dynamics of filamentous/colonial cyanobacteria are consistent with observations in the Gulf of Finland, NW Baltic Proper and South Eastern Baltic Sea (Wasmund et al., 2001; Laamanen and Kuosa, 2005; Walve and Larsson, 2007). Previously, Suikkanen et al. (2010) proposed a conceptual model of different life-cycle strategies of cyanobacteria in the Baltic Sea. The model builds on the assumption that *Aphanizomenon* is present all year in the upper mixed layer while *Dolichospermum* overwinter in the sediment, and *Nodularia* can overwinter both in the water column and the sediment. Our results support this assumption since *Aphanizomenon* remained in the surface mixed layer over a wide range of temperature and nutrient conditions, and peaked at the most favorable conditions during summer. Further, we hypothesize that *Dolichospermum* and *Nodularia* are likely overwintering in the sediment and proliferate in the upper mixed layer upon mixing events during stratification. Still, there was no evidence of *Nodularia* overwintering in the water column in contrast to the model of Suikkanen et al. (2010). This could be due to (i) different mixing patterns between the Gulf of Finland and the Western Gotland Sea (this study) or the Northern Baltic Sea (Hajdu et al., 2007), (ii) vertical transport of nutrients (Wasmund et al., 2012), (iii) varying life cycle strategies of different populations and (iv) different sampling frequency and resolution.

Potential toxic species such as *Nodularia* and *Dolichospermum* were more abundant during the summer bloom in 2011 (**Figure 1**) when stratification was established in late June–July, i.e., later than in 2010 (Legrand et al., 2015). As those species were not found in the water column until early summer, we suggest that in future climate conditions, the recruitment of these toxic cyanobacteria to the water column will strongly depend on mixing events and not solely on low N:P ratios (Nausch et al., 2008) and high temperature.

### Cyanobacterial Phylogeny

The Baltic Sea supports high phylogenetic diversity of filamentous/colonial (**Figure 2**), and picocyanobacteria (Sánchez-Baracaldo et al., 2008, **Figure 2**). Five genera of filamentous/colonial cyanobacteria (*Aphanizomenon/Dolichospermum, N. spumigena, Pseudanabaena, Planktothrix, Snowella*) were identified, confirming the common bloom-forming species in the Baltic Sea (Stal et al., 2003, **Figure 1**). All *N. spumigena* OTUs (22 OTUs) clustered in one major group, exhibiting high phylogentic diversity (data not shown). This is in contrast with previous studies where a single genotype was found to dominate the *Nodularia* population during the summer (Smith et al., 1993; Stal et al., 2003). However, a direct comparison with these studies from 1993 and 2003 is problematic given the different sequencing techniques and the statistical population sizes (i.e., the number of reads). *Aphanizomenon* and *Dolichospermum* OTUs showed many distinct clusters (**Figure 2**). However, these two cyanobacteria have a close phylogenetic relationship and they cannot be defined as different genera using 16S rRNA gene sequence analysis (Lyra et al., 2001; Gugger et al., 2002; Rajaniemi et al., 2005; Stüken et al., 2009). Taxonomical resolution of these two genera can be obtained using the phycocyanin operon as marker (PC-IGS; Janson and Granéli, 2002) or microscopy. These results indicate a lower genetic diversity than compared to observations of the phenotypes alone (*Aphanizomenon* and *Dolichospermum*), and support the importance of using a polyphasic approach (genotypes and phenotypes, Willame et al., 2006) to obtain a comprehensive and accurate description of cyanobacterial community composition in the Baltic Sea.

Among the most abundant cyanobacterial OTUs, more than half (46 OTUs) belonged to the picocyanobacteria *Synechococcus/Cyanobium* (**Figure 2**) illustrating the high phylogenetic diversity within this primary producer group (Haverkamp et al., 2008; Ahlgren and Rocap, 2012). Picocyanobacteria have greater phylogenetic diversity in freshwater than in marine systems (Sánchez-Baracaldo et al., 2008). In our study, the majority of the picocyanobacteria phylotypes were related to non-marine strains, obtained from fresh to brackish waters (**Figure 2**). A high phylogenetically diverse consortium of picocyanobacteria is present in the Baltic Sea, and the number of new OTUs and clusters reported here is staggering, yet varied depending on sampling location. This large phylogenetic diversity within *Synechococcus/Cyanobium* supports the ability of this taxon to thrive in dynamic environments and may explain the year-round presence of these single cells within the cyanobacteria community despite seasonal changes.

### Community Sructure: Generalists vs. Opportunists

Recent studies on prokaryotic diversity and community composition in the Baltic Sea have considered all cyanobacteria (filamentous/colonial and single-celled picocyanobacteria) as one group (Dupont et al., 2014; Lindh et al., 2015). This grouping fails to disentangle the different strategies within and among cyanobacterial phenotypes/genera for seasonal succession and adaptation to environmental changes. In our study, two clear strategies concerning the occurrence of cyanobacteria were found under different environmental conditions: the emergence of (i) OTUs/clusters in all samples and all stations (i.e., generalist) and (ii) several highly seasonal OTUs/clusters (i.e., opportunists).

The most abundant OTU within this generalist cluster in filamentous/colonial cyanobacteria was highly related (>99% 16S rRNA) to previously reported sequences (Casero et al., 2014). Despite this, the physiology of this prominent OTU is largely unknown. The dominance of this cluster over 2 years regardless of the presence of other clusters/OTUs (**Figure 3**) suggests an epidemic population structure of *Aphanizomenon/Dolichospermum* similar to *Planktothrix* (D’Alelio et al., 2013). By contrast, opportunists, here exemplified with *N. spumigena*, are clonal and do not show an epidemic population structure unlike the hypothesis suggested by Smith et al. (1993) and Stal et al. (2003). The proliferation of these opportunistic OTUs relies on recruitment either from the water column or the sediment as discussed above. The fact that these phylotypes co-occur suggests that they do not compete for resources (nutrients) and implicate that they have distinct ecological niches related to temperature, salinity, and nutrients.

Studies of seasonal dynamics of picocyanobacteria in the Southern California Bight and in Chesapeake Bay have shown the co-occurrence of generalists (Clades 1 and 4) and opportunistic (Clades 2 and 3) *Synechococcus* (Tai and Palenik, 2009; Cai et al., 2010). In the Baltic Sea, seasonal patterns in microbial community dynamics, including cyanobacteria, were observed in studies focusing on heterotrophic bacteria (Andersson et al., 2010; Lindh et al., 2015). Our study revealed, for the first time, that few generalists and many opportunistic OTUs were present among picocyanobacteria. Generally, opportunistic picocyanobacteria occurred tightly coupled to the opportunistic filamentous cyanobacteria, mostly after they have bloomed (**Figure 3** and **Supplementary Figure S4**). A deeper analysis of the data on bacterial population dynamics presented by Lindh et al. (2015; **Figure 3**) also showed two distinct occurrence patterns among the cyanobacteria, corresponding to opportunistic filamentous/colonial OTUs in summer and opportunistic picocyanobacteria OTUs in late summer/fall. This implies a strong seasonal succession of communities both filamentous and picocyanobacteria in the Baltic Sea, likely explained by environmental dynamics but also by interactions between these two groups of cyanobacteria.

### Community Structure: Spatial, Temporal, and Environmental Dynamics

The structure of marine microbial communities responds to spatial and temporal variability (Fuhrman et al., 2015). However, large geographical distances play a secondary role in shaping microbial communities (Sunagawa et al., 2015). Our results revealed only minor spatial differences in cyanobacterial community structure between coastal and offshore waters over the 100 km^2^ sampled area (**Figure 4**). Temperature, salinity, stability of the water mass and availability of N and P are the main factors controlling cyanobacterial communities in the Baltic Sea (Larsson et al., 1985; Wasmund et al., 2012; Andersson et al., 2015). In our study area, winter temperatures and spring bloom intensity were correlated to the magnitude of the cyanobacteria bloom in summer (Legrand et al., 2015). It is well known that microbial community composition changes along the longitudinal salinity gradient in the Baltic Sea, as observed from the study of summer transects (Herlemann et al., 2011; Dupont et al., 2014). In our seasonal study, both temperature and salinity were two potential factors shaping cyanobacteria community structure. In particular, the temporal patterns of the opportunistic genera e.g., *Nodularia* and *Pseudanabaena* were driven by changes in salinity likely associated to a disturbance in the environment e.g., winds. Additionally, opportunistic picocyanobacteria occurrence has been mainly explained by changes in environmental conditions (temperature, salinity, and nutrients). It is likely that a large proportion of the changes in microbial community structure can mainly be explained by unknown factors (Souffreau et al., 2015). Considering that filamentous/colonial cyanobacteria can produce bioactive compounds (Mazur-Marzec et al., 2013a) with antibacterial properties (Legrand et al., 2003), chemical interactions in microbial communities can play an important role in community composition.

Conventionally, the decline of filamentous and colonial cyanobacteria bloom is attributed to phosphate limitation (Walve and Larsson, 2007). In our study, many opportunistic cyanobacteria, including N_2_-fixers and picocyanobacteria, were present when levels of both TN and TP were low (**Figure 6**). Here we highlight the burst in diversity and abundance of picocyanobacteria corresponding to the onset of the bloom decline of filamentous/colonial cyanobacteria (**Figure 3**), possibly taking advantage of the bioavailable N fuelled into the system by heterocystous N_2_-fixers (*Aphanizomenon*, *Dolichospermum*, and *Nodularia*; Ploug et al., 2011). Higher diversity of picocyanobacteria might be related, rather than to nitrogen, to other compounds produced by opportunistic filamentous cyanobacteria. In addition, phosphorus remineralization by heterotrophic bacteria can exceed their P demand (White et al., 2012) and can be an extra source of P for autotrophs even at low ambient P concentrations.

### Cyanobacterial Communities and Future Climate Conditions

Levels of community specialization could be an indicator of the impact of global changes (environmental disturbances) on community structure (Clavel et al., 2011), suggesting that changes in environmental conditions related to climate change may promote a shift toward communities being dominated by generalists. Alternatively, it is also possible that future environmental shifts related to climate change induce more favorable conditions for opportunists. Our study suggests that in addition to temperature, short-term shifts in salinity can potentially shape cyanobacterial community structure. Since regional climate scenarios predict both higher temperature and lower salinity as future climate conditions in the Baltic Sea (Meier et al., 2014), we propose that these future environmental conditions could provide opportunistic filamentous/colonial cyanobacteria with a competitive advantage in the planktonic habitat. Further experimental trials are necessary to confirm that combined low salinity and high temperature would benefit opportunistic filamentous/colonial cyanobacteria. This would be paramount for assessing potential effects of cyanobacteria on other trophic levels and ultimately on the status of the Baltic Sea ecosystems.

## Author Contributions

CL, JP, MC, and AA conceived the study. MB-F, HF, ML, MC, and CL designed the research. MB-F, ML, MC, and CL organized the fieldwork and performed the sampling. MB-F and ML performed molecular work. MB-F, HF, and ML analyzed the data and CL and JP helped with data interpretation. MB-F, HF, and CL wrote the manuscript and all authors discussed the results and commented on the manuscript.

## Conflict of Interest Statement

The authors declare that the research was conducted in the absence of any commercial or financial relationships that could be construed as a potential conflict of interest.

## Footnotes

1 http://nordicmicroalgae.org/

## Supplementary Material

**FIGURE S1 | Bathymetric map of the study site and sampling stations.** **FIGURE S2 | Rarefaction curves of the number of observed cyanobacterial OTUs at 98% identity cut-off.** **FIGURE S3 | Spatial and temporal variation of temperature, salinity and Chl *a* concentration over 2010–2011 and total nitrogen, total phosphorus, dissolved inorganic nitrogen, phosphate, TN/TP ratio during 2011 in the euphotic zone (10 m).** Data adapted from Legrand et al. (2015). **FIGURE S4 | Number of OTUs per station affiliated to filamentous/colonial and picocyanobacteria in year 2010 and 2011.** **FIGURE S5 | Relative abundance of the most frequently occurring OTUs within filamentous cyanobacteria **(A)** OTU000006 (*Aphanizomenon/**Dolichospermum*), **(B)** OTU000113 (*Nodularia spumigena*); and picocyanobacteria **(C)** OTU000001, **(D)** OTU000034, **(E)** OTU000072 (*Synechococcus*).**

## References

[B1] Ahlgren N. A. Rocap G. (2012). Diversity and distribution of marine *Synechococcus*: multiple gene phylogenies for consensus classification and development of qPCR assays for sensitive measurement of clades in the ocean. *Front. Microbiol.* 3:213 10.3389/fmicb.2012.00213 PMC3377940 22723796

[B2] Andersson A. Höglander H. Karlsson C. Huseby S. (2015). Key role of phosphorus and nitrogen in regulating cyanobacterial community composition in the northern Baltic Sea. *Estuar. Coast. Shelf Sci.* 164 161–171. 10.1016/j.ecss.2015.07.013

[B3] Andersson A. F. Riemann L. Bertilsson S. (2010). Pyrosequencing reveals contrasting seasonal dynamics of taxa within Baltic Sea bacterioplankton communities. *ISME J.* 4 171–181. 10.1038/ismej.2009.108 19829318

[B4] Barker G. L. A. Hayes P. K. Mahony S. L. O. Vacharapiyasophon P. Walsby A. E. Ku N. (1999). A molecular and phenotypic analysis of *Nodularia* (Cyanobacteria) from the Baltic Sea. *J. Phycol.* 35 931–937. 10.1046/j.1529-8817.1999.3550931.x

[B5] Bianchi T. S. Engelhaupt E. Westman P. Andren T. Rolff C. (2000). Cyanobacterial blooms in the Baltic Sea : natural or human-induced? *Limnol. Oceanogr.* 45 716–726. 10.4319/lo.2000.45.3.0716

[B6] Cai H. Wang K. Huang S. Jiao N. Chen F. (2010). Distinct patterns of picocyanobacterial communities in winter and summer in the Chesapeake Bay. *Appl. Environ. Microbiol.* 76 2955–2960. 10.1128/AEM.02868-09 20228109 PMC2863441

[B7] Casero M. C. Ballot A. Agha R. Quesada A. Cirés S. (2014). Characterization of saxitoxin production and release and phylogeny of sxt genes in paralytic shellfish poisoning toxin-producing *Aphanizomenon gracile*. *Harmful Algae* 37 28–37. 10.1016/j.hal.2014.05.006

[B8] Clavel J. Julliard R. Devictor V. (2011). Worldwide decline of specialist species: toward a global functional homogenization? *Front. Ecol. Environ.* 9:222–228 10.1890/080216

[B9] Crosbie N. D. Pöckl M. Weisse T. (2003). Dispersal and phylogenetic diversity of nonmarine picocyanobacteria, inferred from 16S rRNA gene and cpcBA -Intergenic Spacer sequence analyses. *Appl. Environ. Microbiol.* 69 5716–5721. 10.1128/AEM.69.9.5716-5721.2003 12957969 PMC194977

[B10] D’Alelio D. Salmaso N. Gandolfi A. (2013). Frequent recombination shapes the epidemic population structure of *Planktothrix* (Cyanoprokaryota) in italian subalpine lakes. *J. Phycol.* 49 1107–1117. 10.1111/jpy.12116 27007631

[B11] Dupont C. L. Larsson J. Yooseph S. Ininbergs K. Goll J. Asplund-Samuelsson J. (2014). Functional tradeoffs underpin salinity-driven divergence in microbial community composition. *PLoS ONE* 9:e89549 10.1371/journal.pone.0089549 PMC3937345 24586863

[B12] Edgar R. C. (2010). Search and clustering orders of magnitude faster than BLAST. *Bioinformatics* 26 2460–2461. 10.1093/bioinformatics/btq461 20709691

[B13] Edler L. (1979). Recommendations on methods for marine biological studies in the Baltic Sea: phytoplankton and chlorophyll. *In Baltic Marine Biologists No* 5 1–38.

[B14] Eriksson J. E. Toivola D. Meriluoto J. A. O. Karaki H. Han Y.-G. Hartshorne D. (1990). Hepatocyte deformation induced by cyanobacterial toxins reflects inhibition of protein phosphatases. *Biochem. Biophys. Res. Commun.* 173 1347–1353. 10.1016/S0006-291X(05)80936-2 2176489

[B15] Fewer D. P. Jokela J. Paukku E. Österholm J. Wahlsten M. Permi P. (2013). New structural variants of aeruginosin produced by the toxic bloom forming cyanobacterium *Nodularia spumigena*. *PLoS ONE* 8:e73618 10.1371/journal.pone.0073618 PMC3765200 24040002

[B16] Fuhrman J. A. Cram J. A. Needham D. M. (2015). Marine microbial community dynamics and their ecological interpretation. *Nat. Rev. Microbiol.* 13 133–146. 10.1038/nrmicro3417 25659323

[B17] Gasol J. M. del Giorgio P. A. (2000). Using flow cytometry for counting natural planktonic bacteria and understanding the structure of planktonic bacterial communities. *Sci. Mar.* 64 197–224.

[B18] Gugger M. Lyra C. Henriksen P. Couté A. Humbert J.-F. Sivonen K. (2002). Phylogenetic comparison of the cyanobacterial genera *Anabaena* and *Aphanizomenon*. *Int. J. Syst. Evol. Microbiol.* 52 1867–1880. 10.1099/00207713-52-5-1867 12361299

[B19] Hajdu S. Höglander H. Larsson U. (2007). Phytoplankton vertical distributions and composition in Baltic Sea cyanobacterial blooms. *Harmful Algae* 6 189–205. 10.1016/j.hal.2006.07.006

[B20] Haverkamp T. Acinas S. G. Doeleman M. Stomp M. Huisman J. Stal L. J. (2008). Diversity and phylogeny of Baltic Sea picocyanobacteria inferred from their ITS and phycobiliprotein operons. *Environ. Microbiol.* 10 174–188. 17903216 10.1111/j.1462-2920.2007.01442.x

[B21] Hense I. Meier H. E. M. Sonntag S. (2013). Projected climate change impact on Baltic Sea cyanobacteria. *Clim. Change* 119 391–406. 10.1007/s10584-013-0702-y

[B22] Herlemann D. P. Labrenz M. Jürgens K. Bertilsson S. Waniek J. J. Andersson A. F. (2011). Transitions in bacterial communities along the 2000 km salinity gradient of the Baltic Sea. *ISME J.* 5 1571–1579. 10.1038/ismej.2011.41 21472016 PMC3176514

[B23] Hoegh-Guldberg O. Bruno J. F. (2010). The impact of climate change on the world’s marine ecosystems. *Science* 328 1523–1528. 10.1126/science.1189930 20558709

[B24] Janson S. Granéli E. (2002). Phylogenetic analyses of nitrogen-fixing cyanobacteria from the Baltic Sea reveal sequence anomalies in the phycocyanin operon. *Int. J. Syst. Evol. Microbiol.* 52 1397–1404. 10.1099/00207713-52-4-1397 12148656

[B25] Jespersen A. M. Christoffersen K. (1987). Measurements of chlorophyll-a from phytoplankton using ethanol as extraction solvent. *Arch. Hydrobiol.* 109 445–454.

[B26] Kahru M. Elmgren R. (2014). Multidecadal time series of satellite-detected accumulations of cyanobacteria in the Baltic Sea. *Biogeosciences* 11 3619–3633. 10.5194/bg-11-3619-2014

[B27] Karjalainen M. Engström-Öst J. Korpinen S. Peltonen H. Pääkkönen J.-P. Rönkkönen S. (2007). Ecosystem consequences of cyanobacteria in the northern Baltic Sea. *Ambio* 36 195–202. 10.1579/0044-7447(2007)36[195:ECOCIT]2.0.CO;2 17520934

[B28] Komárek J. Kaštovský J. Mareš J. Johansen J. (2014). Taxonomic classification of cyanoprokaryotes (cyanobacterial genera) 2014 using a polyphasic approach. *Preslia* 86 295–335.

[B29] Laamanen M. Kuosa H. (2005). Annual variability of biomass and heterocysts of the N2-fixing cyanobacterium *Aphanizomenon flos-aquae* in the Baltic Sea with reference to *Anabaena* spp. and *Nodularia spumigena*. *Boreal Environ. Res.* 10 19–30.

[B30] Larsson J. Celepli N. Ininbergs K. Dupont C. L. Yooseph S. Bergman B. (2014). Picocyanobacteria containing a novel pigment gene cluster dominate the brackish water Baltic Sea. *ISME J.* 8 1892–1903. 10.1038/ismej.2014.35 24621524 PMC4139726

[B31] Larsson U. Elmgren R. Wulff F. (1985). Eutrophication and the Baltic Sea: causes and consequences. *Ambio* 14 10–14.

[B32] Legrand C. Fridolfsson E. Bertos-Fortis M. Lindehoff E. Larsson P. Pinhassi J. (2015). Interannual variability of phyto-bacterioplankton biomass and production in coastal and offshore waters of the Baltic Sea. *Ambio* 44 427–438. 10.1007/s13280-015-0662-8 26022325 PMC4447688

[B33] Legrand C. Rengefors K. Fistarol G. O. Graneli E. (2003). Allelopathy in phytoplankton – biochemical, ecological and evolutionary aspects. *Phycologia* 42 406–419. 10.2216/i0031-8884-42-4-406.1

[B34] Lindh M. V. Sjöstedt J. Andersson A. F. Baltar F. Hugerth L. W. Lundin D. (2015). Disentangling seasonal bacterioplankton population dynamics by high-frequency sampling. *Environ. Microbiol.* 17 2459–2476. 10.1111/1462-2920.12720 25403576

[B35] Lips I. Lips U. (2008). Abiotic factors influencing cyanobacterial bloom development in the Gulf of Finland (Baltic Sea). *Hydrobiologia* 614 133–140. 10.1007/s10750-008-9449-2

[B36] Lyra C. Suomalainen S. Gugger M. Vezie C. Sundman P. Paulin L. (2001). Molecular characterization of planktic cyanobacteria of *Anabaena*, *Aphanizomenon*, *Microcystis* and *Planktothrix* genera. *Int. J. Syst. Evol. Microbiol.* 51 513–526. 10.1099/00207713-51-2-513 11321098

[B37] Mazur-Marzec H. Kaczkowska M. J. Blaszczyk A. Akcaalan R. Spoof L. Meriluoto J. (2013a). Diversity of peptides produced by *Nodularia spumigena* from various geographical regions. *Mar. Drugs* 11 1–19. 10.3390/md11010001 23344154 PMC3564153

[B38] Mazur-Marzec H. Sutryk K. Kobos J. Hebel A. Hohlfeld N. Błaszczyk A. (2013b). Occurrence of cyanobacteria and cyanotoxin in the Southern Baltic Proper. Filamentous cyanobacteria versus single-celled picocyanobacteria. *Hydrobiologia* 701 235–252. 10.1007/s10750-012-1278-7

[B39] Meier H. E. M. Andersson H. Arheimer B. Donnelly C. Eilola K. Gustafsson B. (2014). Ensemble modeling of the Baltic Sea ecosystem to provide scenarios for management. *Ambio* 43 37–48. 10.1007/s13280-013-0475-6 24414803 PMC3888662

[B40] Nausch M. Nausch G. Wasmund N. Nagel K. (2008). Phosphorus pool variations and their relation to cyanobacteria development in the Baltic Sea: a three-year study. *J. Mar. Syst.* 71 99–111. 10.1016/j.jmarsys.2007.06.004

[B41] Niemi Å (1979). Blue-green algal blooms and N:P ratio in the Baltic Sea. *Acta Bot. Fenn.* 110 57–61.

[B42] Ohta T. Sueoka E. Iida N. Komori A. Suganuma M. Nishiwaki R. (1994). Nodularin, a potent inhibitor of protein phosphatase-1 and phosphatase-2A, is a new environmental carcinogen in male F344 rat-liver. *Cancer Res.* 54 6402–6406. 7527297

[B43] Oksanen J. Blanchet F. G. Kindt R. Legendre P. Minchin P. R. O’Hara R. B. (2016). *Vegan: Community Ecology Package. R Package Version 2.0–10*. Available at: http://CRAN.R-project.org/package=vegan

[B44] Olenina I. Hajdu S. Edler L. Andersson A. Wasmund N. Busch S. (2006). *Biovolumes and Size-Classes of Phytoplankton in the Baltic Sea. In HELCOM Baltic Sea Environment Proceedings No. 106 144*. Available at: http://helcom.fi/Lists/Publications/BSEP106.pdf

[B45] Paerl H. W. Huisman J. (2009). Climate change: a catalyst for global expansion of harmful cyanobacterial blooms. *Environ. Microbiol. Rep.* 1 27–37. 10.1111/j.1758-2229.2008.00004.x 23765717

[B46] Ploug H. Adam B. Musat N. Kalvelage T. Lavik G. Wolf-Gladrow D. (2011). Carbon, nitrogen and O2 fluxes associated with the cyanobacterium *Nodularia spumigena* in the Baltic Sea. *ISME J.* 5 1549–1558. 10.1038/ismej.2011.20 21390075 PMC3160678

[B47] Quince C. Lanzen A. Davenport R. J. Turnbaugh P. J. (2011). Removing noise from pyrosequenced amplicons. *BMC Bioinformatics* 12:38 10.1186/1471-2105-12-38 PMC3045300 21276213

[B48] Rajaniemi P. Hrouzek P. Kastovská K. Willame R. Rantala A. Hoffmann L. (2005). Phylogenetic and morphological evaluation of the genera *Anabaena*, *Aphanizomenon*, *Trichormus* and *Nostoc* (Nostocales, Cyanobacteria). *Int. J. Syst. Evol. Microbiol.* 55 11–26. 10.1099/ijs.0.63276-0 15653847

[B49] Riemann L. Steward G. F. Azam F. (2000). Dynamics of bacterial community composition and activity during a mesocosm diatom bloom. *Appl. Environ. Microbiol.* 66 578–587. 10.1128/AEM.66.2.578-587.2000 10653721 PMC91866

[B50] Sanchez G. (2015). *Partial Least Squares (PLS) Data Analysis Methods. R Package Version 0.1.17*. Available at: http://cran.r-project.org/web/packages/plsdepot/index.html

[B51] Sánchez-Baracaldo P. Handley B. A. Hayes P. K. (2008). Picocyanobacterial community structure of freshwater lakes and the Baltic Sea revealed by phylogenetic analyses and clade-specific quantitative PCR. *Microbiology* 154 3347–3357. 10.1099/mic.0.2008/019836-0 18957588

[B52] Smith J. M. Smith N. H. O’Rourke M. Spratt B. G. (1993). How clonal are bacteria? *Proc. Natl. Acad. Sci. U.S.A.* 90 4384–4388. 10.1073/pnas.90.10.4384 8506277 PMC46515

[B53] Souffreau C. Van der Gucht K. van Gremberghe I. Kosten S. Lacerot G. Lobão L. M. (2015). Environmental rather than spatial factors structure bacterioplankton communities in shallow lakes along a > 6000 km latitudinal gradient in South America. *Environ. Microbiol.* 17 2336–2351. 10.1111/1462-2920.12692 25471233

[B54] Stal L. J. Albertano P. Bergman B. Bröckel K. Von Gallon J. R. Hayes P. K. (2003). BASIC: Baltic Sea cyanobacteria. An investigation of the structure and dynamics of water blooms of cyanobacteria in the Baltic Sea—responses to a changing environment. *Cont. Shelf Res.* 23 1695–1714. 10.1016/j.csr.2003.06.001

[B55] Stüken A. Campbell R. J. Quesada A. Sukenik A. Dadheech P. K. Wiedner C. (2009). Genetic and morphologic characterization of four putative cylindrospermopsin producing species of the cyanobacterial genera *Anabaena* and *Aphanizomenon*. *J. Plankton Res.* 31 465–480. 10.1093/plankt/fbp011

[B56] Suikkanen S. Kaartokallio H. Hällfors S. Huttunen M. Laamanen M. (2010). Life cycle strategies of bloom-forming, filamentous cyanobacteria in the Baltic Sea. *Deep Sea Res. Part II Top. Stud. Oceanogr.* 57 199–209. 10.1016/j.dsr2.2009.09.014

[B57] Sunagawa S. Coelho L. P. Chaffron S. Kultima J. R. Labadie K. Salazar G. (2015). Structure and function of the global ocean microbiome. *Science* 348 1–9. 10.1126/science.1261359 25999513

[B58] Tai V. Palenik B. (2009). Temporal variation of *Synechococcus* clades at a coastal Pacific Ocean monitoring site. *ISME J.* 3 903–915. 10.1038/ismej.2009.35 19360028

[B59] Tamura K. Stecher G. Peterson D. Filipski A. Kumar S. (2013). MEGA6: molecular evolutionary genetics analysis version 6.0. *Mol. Biol. Evol.* 30 2725–2729. 10.1093/molbev/mst197 24132122 PMC3840312

[B60] Urbach E. Scanlan D. J. Distel D. L. Waterbury J. B. Chisholm S. W. (1998). Rapid diversification of marine picophytoplankton with dissimilar light– harvesting structures inferred from sequences of *Prochlorococcus* and *Synechococcus* (cyanobacteria). *J. Mol. Evol.* 46 188–201. 10.1007/PL00006294 9452521

[B61] Vahtera E. Conley D. J. Gustafsson B. G. Kuosa H. Pitkänen H. Savchuk O. P. (2007). Internal ecosystem feedbacks enhance nitrogen-fixing cyanobacteria blooms and complicate management in the Baltic Sea. *Ambio* 36 186–194. 10.1579/0044-7447(2007)36[186:IEFENC]2.0.CO;2 17520933

[B62] Valderrama J. (1995). “Methods of nutrient analysis,” in *Manual of Harmful Marine Microalgae: IOC Manuals and Guides* Vol. 33 eds Hallegraeff G. M. Anderson D. M. Cembella A. D. (Paris: UNESCO), 251–268.

[B63] Wacklin P. Hoffmann L. Komárek J. (2009). Nomenclatural validation of the genetically revised cyanobacterial genus *Dolichospermum* (Ralfs ex Bornet et Flahault). *Fottea* 9 59–64. 10.5507/fot.2009.005

[B64] Walve J. Larsson U. (2007). Blooms of Baltic Sea *Aphanizomenon* sp. (Cyanobacteria) collapse after internal phosphorus depletion. *Aquat. Microb. Ecol.* 49 57–69. 10.3354/ame01130

[B65] Wasmund N. Andrushaitis A. Łysiak-Pastuszak E. Müller-Karulis B. Nausch G. Neumann T. (2001). Trophic status of the south-eastern Baltic Sea: a comparison of coastal and open areas. *Estuar. Coast. Shelf Sci.* 53 849–864. 10.1006/ecss.2001.0828

[B66] Wasmund N. Nausch G. Voss M. (2012). Upwelling events may cause cyanobacteria blooms in the Baltic Sea. *J. Mar. Syst.* 90 67–76. 10.1016/j.jmarsys.2011.09.001

[B67] Waterbury J. B. Watson S. W. Valois F. W. Franks D. G. (1986). Biological and ecological characterization of the marine unicellular cyanobacterium *Synechococcus*. *Can. Bull. Fish. Aquat. Sci.* 214 71–120.

[B68] White A. E. Watkins-Brandt K. S. Engle M. A. Burkhardt B. Paytan A. (2012). Characterization of the rate and temperature sensitivities of bacterial remineralization of dissolved organic phosphorus compounds by natural populations. *Front. Microbiol.* 3:276 10.3389/fmicb.2012.00276 PMC3415674 22908008

[B69] Willame R. Boutte C. Grubisic S. Wilmotte A. Komárek J. Hoffmann L. (2006). Morphological and molecular characterization of planktonic cyanobacteria from Belgium and Luxembourg. *J. Phycol.* 42 1312–1332. 10.1111/j.1529-8817.2006.00284.x

[B70] Zillén L. Conley D. J. (2010). Hypoxia and cyanobacteria blooms – are they really natural features of the late Holocene history of the Baltic Sea? *Biogeosciences* 7 2567–2580. 10.5194/bg-7-2567-2010

