# Supplementary material for: Unscrambling Cyanobacteria Community Dynamics Related to Environmental Factors
Source: Front Microbiol. 2016 May 9;7:625. doi: 10.3389/fmicb.2016.00625 (PMC4860504; doi:10.3389/fmicb.2016.00625)
Supplement: Supplementary file 1 [file Data_Sheet_1.PDF]

frontiers

Supplementary Material

Unscrambling cyanobacteria community dynamics related to environmental factors

Mireia Bertos-Fortis, Hanna M. Farnelid, Markus V. Lindh, Michele Casini, Agneta Andersson, Jarone Pinhassi and Catherine Legrand*

*** Correspondence:** Catherine Legrand: catherine.legrand@lnu.se

1 Supplementary Figures and Tables

1.1 Supplementary Figures

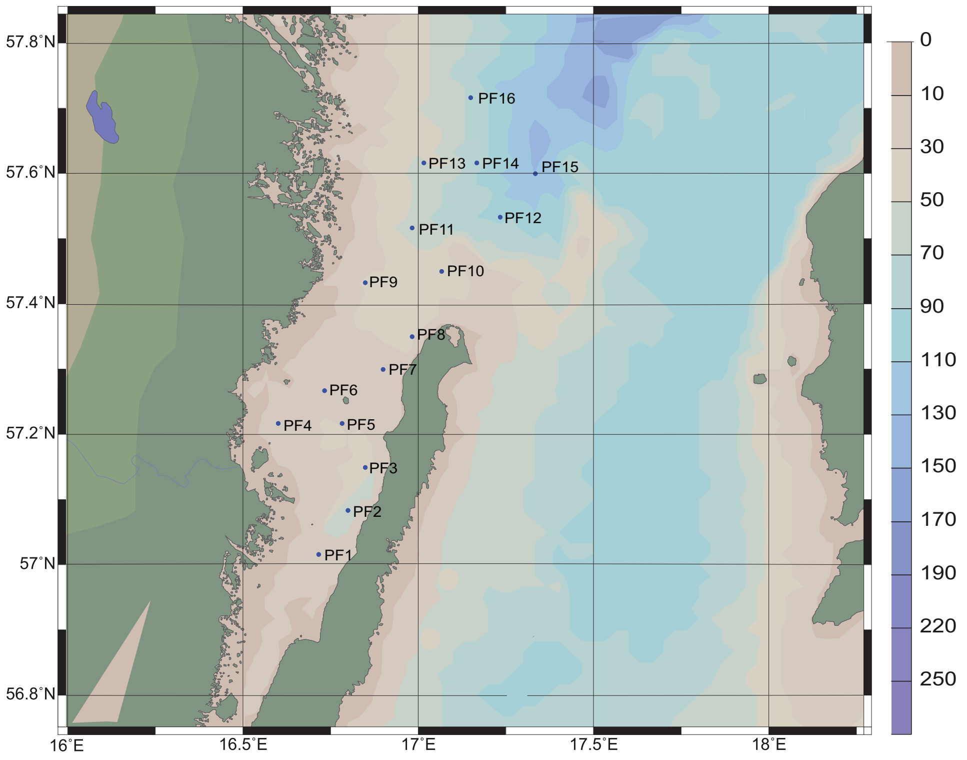

Supplementary Figure 1. Bathymetric map of the study site and sampling stations.

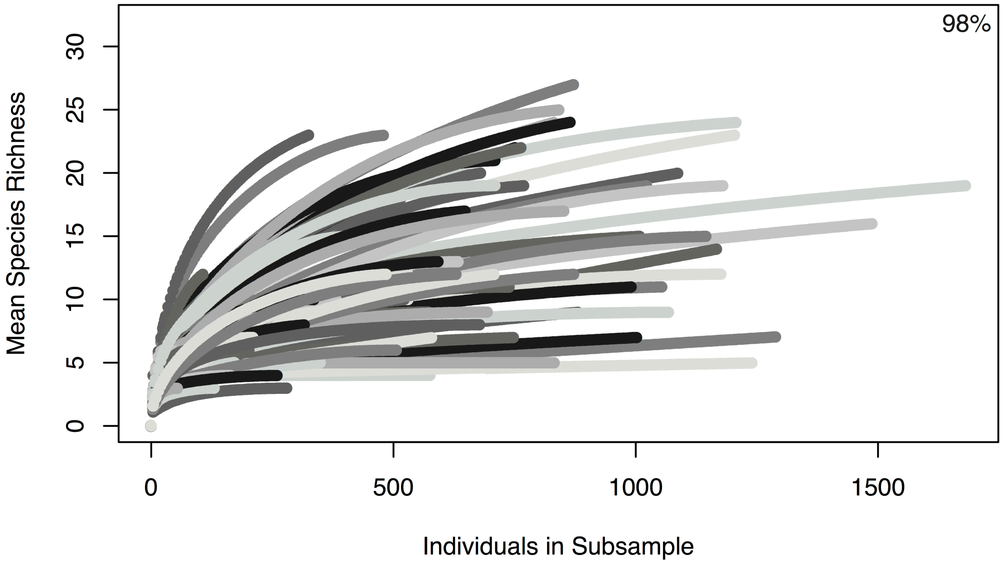

Supplementary Figure 2. Rarefaction curves of the number of observed cyanobacterial OTUs at 98% identity cut-off.

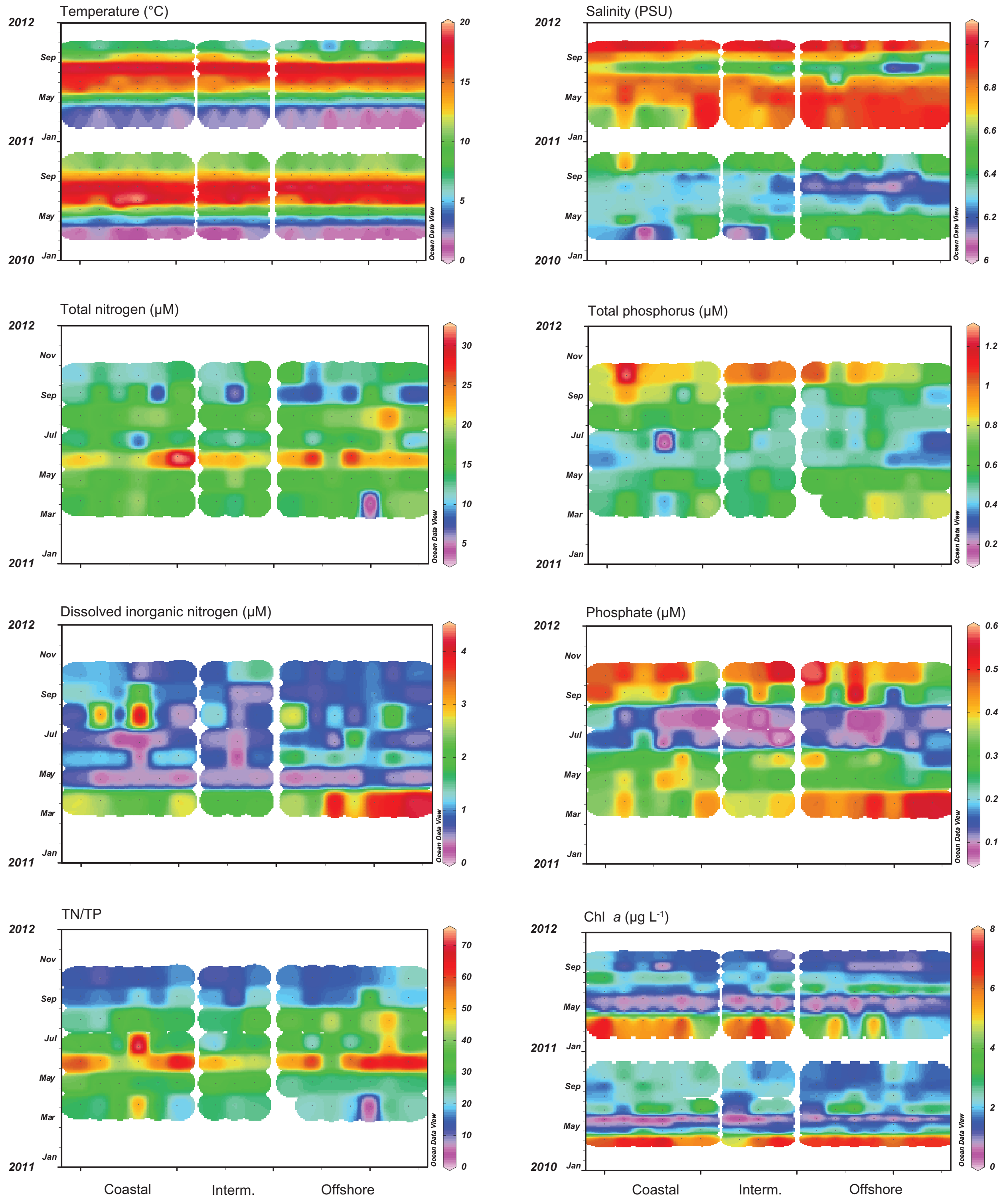

Supplementary Figure 3. Spatial and temporal variation of temperature, salinity and Chl *a* concentration over 2010–2011 and total nitrogen, total phosphorus, dissolved inorganic nitrogen, phosphate, TN/TP ratio during 2011 in the euphotic zone (10 m). Data adapted from Legrand et al. (2015).

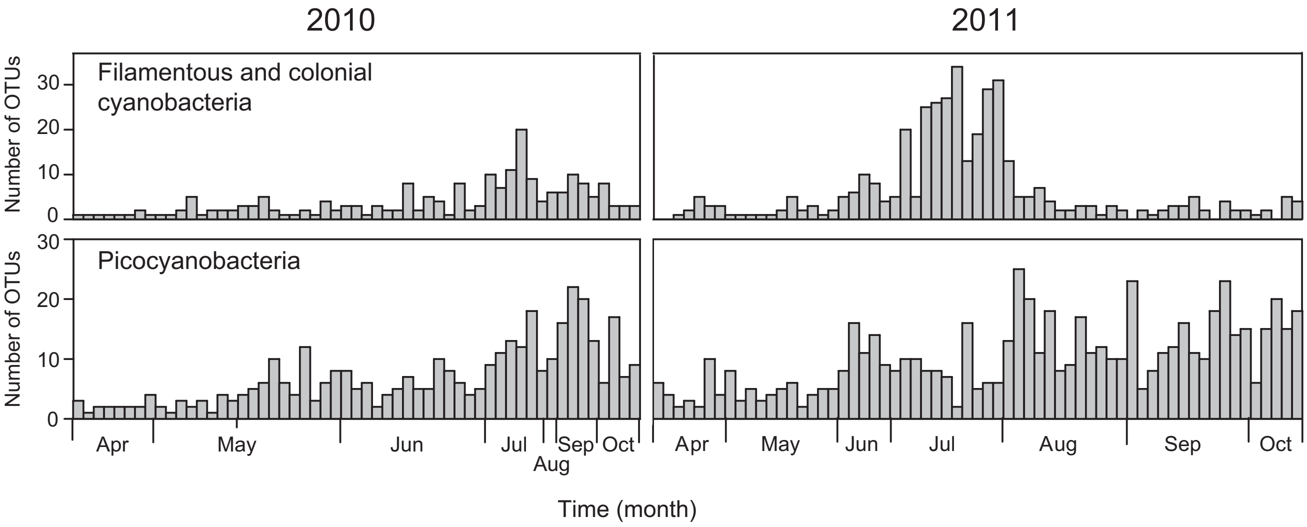

Supplementary Figure 4. Number of OTUs per station affiliated to filamentous/colonial and picocyanobacteria in year 2010 and 2011.

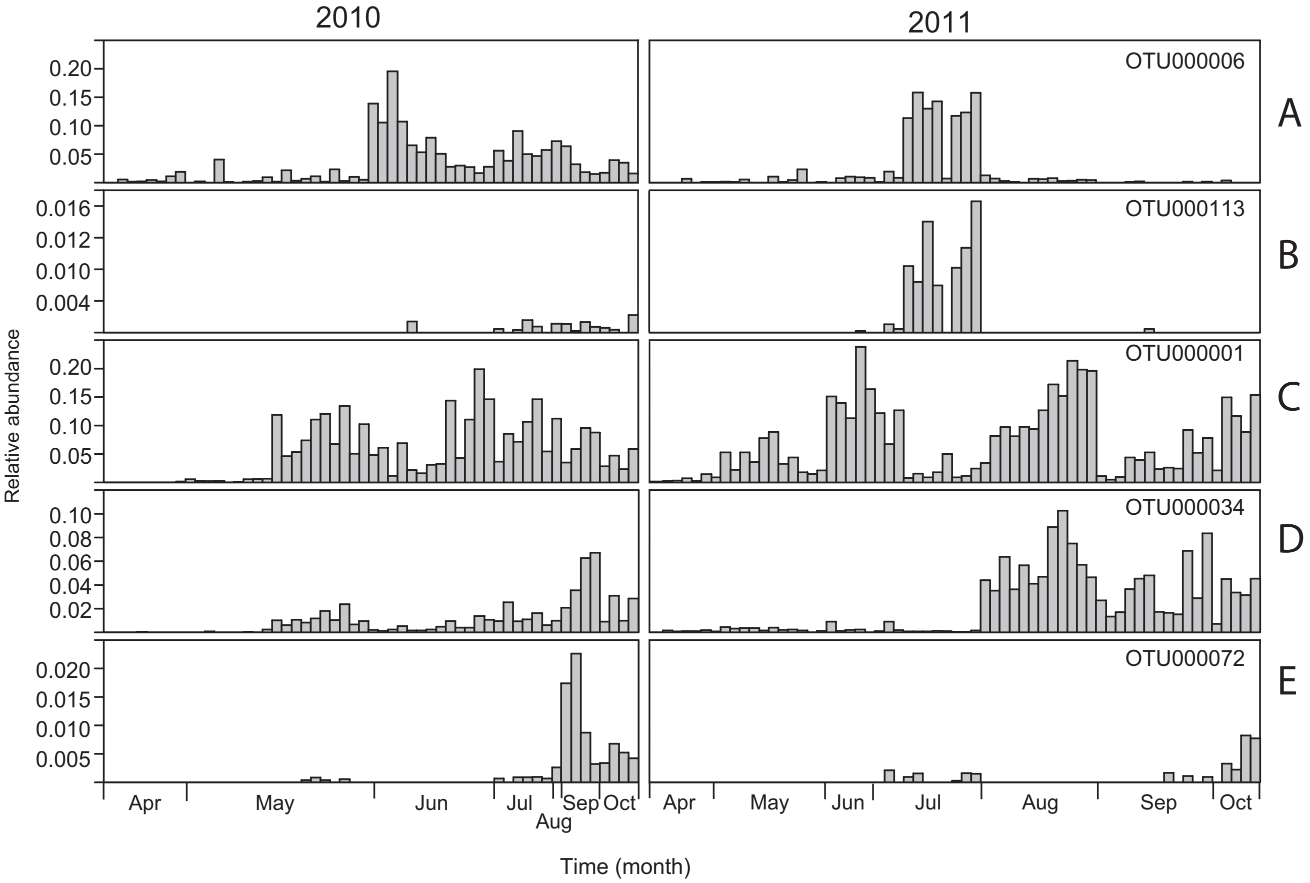

Supplementary Figure 5. Relative abundance of the most frequently occurring OTUs within filamentous cyanobacteria (A) OTU000006 (*Aphanizomenon/Dolichospermum*), (B) OTU000113 (*Nodularia spumigena*); and picocyanobacteria (C) OTU000001, (D) OTU000034, (E) OTU000072 (*Synechococcus*).

1.2 Supplementary Tables

Supplementary Table 1. Table of cumulated Q^2 indexes for PLS components selected by cross-validation in A) Cyanobacterial community composition (100 most abundant OTUs) in 2010 and 2011 related to temperature, salinity, Chl *a* and heterotrophic bacterial abundance B) Cyanobacterial community composition (50 most abundant OTUs) in 2011 related to Temperature, Salinity, Chl *a*, heterotrophic bacterial abundance, TN and TP.

A)

	Q^2cum
t1	0.07
t2	0.03

B)

	Q^2cum
t1	0.35
t2	0.36
t3	0.36

Supplementary Table 2. Impact of temperature and salinity on cyanobacterial community composition. A) Cyanobacterial community composition (100 most abundant OTUs) in 2010 and 2011 related to temperature, salinity B) Cyanobacterial community composition (50 most abundant OTUs) in 2011 related to temperature, salinity.

	A			B		
Factors	F	R^2	*p*	F	R^2	*p*
Temperature	24.1	0.18	<0.001	12.9	0.18	<0.001
Salinity	3.9	0.04	<0.01	4.1	0.06	<0.01
